# Evaluation of two lead malaria transmission blocking vaccine candidate antibodies in natural parasite-vector combinations

**DOI:** 10.1038/s41598-017-06130-1

**Published:** 2017-07-28

**Authors:** Anais Bompard, Dari F. Da, Rakiswendé S. Yerbanga, Sumi Biswas, Melissa Kapulu, Teun Bousema, Thierry Lefèvre, Anna Cohuet, Thomas S. Churcher

**Affiliations:** 1 MRC Centre for Outbreak Analysis and Modelling, Infectious Disease Epidemiology, Imperial College London, Norfolk Place, London, W2 1PG United Kingdom; 2 Institut de Recherche en Sciences de la Santé, Bobo-Dioulasso, Burkina Faso; 3 Unité MIVEGEC, IRD 224-CNRS 5290-Université Montpellier, Montpellier, France; 4 The Jenner Institute, University of Oxford, Oxford, United Kingdom; 5 Department of Medical Microbiology, Nijmegen, The Netherlands; 6 Laboratoire mixte international sur les vecteurs (LAMIVECT), Bobo Dioulasso, Burkina Faso

## Abstract

Transmission blocking vaccines (TBV) which aim to control malaria by inhibiting human-to-mosquito transmission show considerable promise though their utility against naturally circulating parasites remains unknown. The efficacy of two lead candidates targeting Pfs25 and Pfs230 antigens to prevent onwards transmission of naturally occurring parasites to a local mosquito strain is assessed using direct membrane feeding assays and murine antibodies in Burkina Faso. The transmission blocking activity of both candidates depends on the level of parasite exposure (as assessed by the mean number of oocysts in control mosquitoes) and antibody titers. A mathematical framework is devised to allow the efficacy of different candidates to be directly compared and determine the minimal antibody titers required to halt transmission in different settings. The increased efficacy with diminishing parasite exposure indicates that the efficacy of vaccines targeting either Pfs25 or Pfs230 may increase as malaria transmission declines. This has important implications for late-stage candidate selection and assessing how they can support the drive for malaria elimination.

## Introduction

Malaria remain one of the world’s major infectious diseases with an estimated number of 212 million clinical cases annually^1^. Transmission of malaria from human to mosquito occurs when a mosquito ingests mature sexual stages of the parasite, the gametocytes, during blood-feeding. Inside the mosquito, gametocytes undergo the sexual reproduction, the zygotes develop into oocysts on the midgut wall and eventually release the infectious sporozoites. There is increasing interest in the use of vaccine which aim to control the disease by interrupting malaria transmission (VIMT), targeting sexual, sporogonic, and/or mosquito antigens (SSM). SSM-VIMT against mosquito-stage parasites can be categorized as those that target parasite antigens expressed before zygote formation (pre-fertilization antigens, such as Pfs230 and Pfs48/45) and those that affect the subsequent development of parasite stages within the mosquito (post-fertilization antigen, such as Pfs25).

Before clinical trials, SSM-VIMT candidates are assessed in the laboratory using the membrane feeding assays (MFA) whereby antibodies specific to the target antigen versus non-specific control antibodies are produced in model animals and added to *Plasmodium-*infected blood, which is then fed to mosquitoes through a membrane. Mosquitoes are allowed to develop oocysts before being dissected. The efficacy of the intervention is then assessed by comparing the infection success among mosquitoes fed with or without the candidate antibodies. Two estimates are used to measure the efficacy of the intervention: the difference in the number of mosquitoes with oocysts (transmission blocking activity, TBA) and in the number of oocysts within the mosquitoes (transmission reducing activity, TRA). It is assumed that mosquitoes with very low numbers of parasites are capable of transmitting malaria^2, 3^ therefore TBA is the most epidemiologically relevant metric.

Evaluation of SSM-VIMT candidates using cultured parasites by Standard Membrane Feeding Assays (SMFA) have shown that TBA varies according to the level of parasite exposure, which can be measured as the mean number of oocyst in control mosquitoes^4, 5^. Parasite exposure depends primarily on gametocytes density in the blood, though many other factors are known to contribute (for example gametocyte maturity^6^, parasite clone diversity^7^, host blood components^8, 9^ and environnement^10^ amongst others) making it difficult to standardize in laboratory systems. This hinders comparison of SSM-VIMT candidates between experiments^11^ and for predicting efficacy in natural settings. TBA and TRA will also vary with antibody titre^12^. This is another challenge for candidate selection, as achievable titers in humans and titer decay across time are expected vary between candidate antigens, delivery platforms and adjuvants^13^. Therefore it is important to understand the 3D relationship between parasite exposure, antibody titre and TBA to be able to predict the efficacy of the SSM-VIMT candidate in different natural settings and devise robust target product profiles to help select the best candidates to take forward to costly clinical trials.

This study evaluates the efficacy of two leading SSM-VIMT candidates against natural infections of *P. falciparum*. The transmission blocking activity of polyclonal antibodies against Pfs230-C and Pfs25 produced in mice were evaluated using Direct Membrane Feeding Assays (DMFAs). Blood from naturally infected gametocyte carriers underwent serum replacement and were fed to the local vector *Anopheles coluzzii*. A range of antibody titers against *P. falciparum* Pfs230-C and Pfs25 antigens were evaluated using blood donated from 21 volunteers providing a range of naturally circulating gametocyte isolates at natural densities^14, 15^. A novel framework is outlined which measures the complex relationship between efficacy, parasite exposure and antibody titer allowing different SSM-VIMT candidates to be rigorously compared.

## Results

### Transmission-blocking efficacies of anti-Pfs230-C and anti-Pfs25

**Figure 1:**
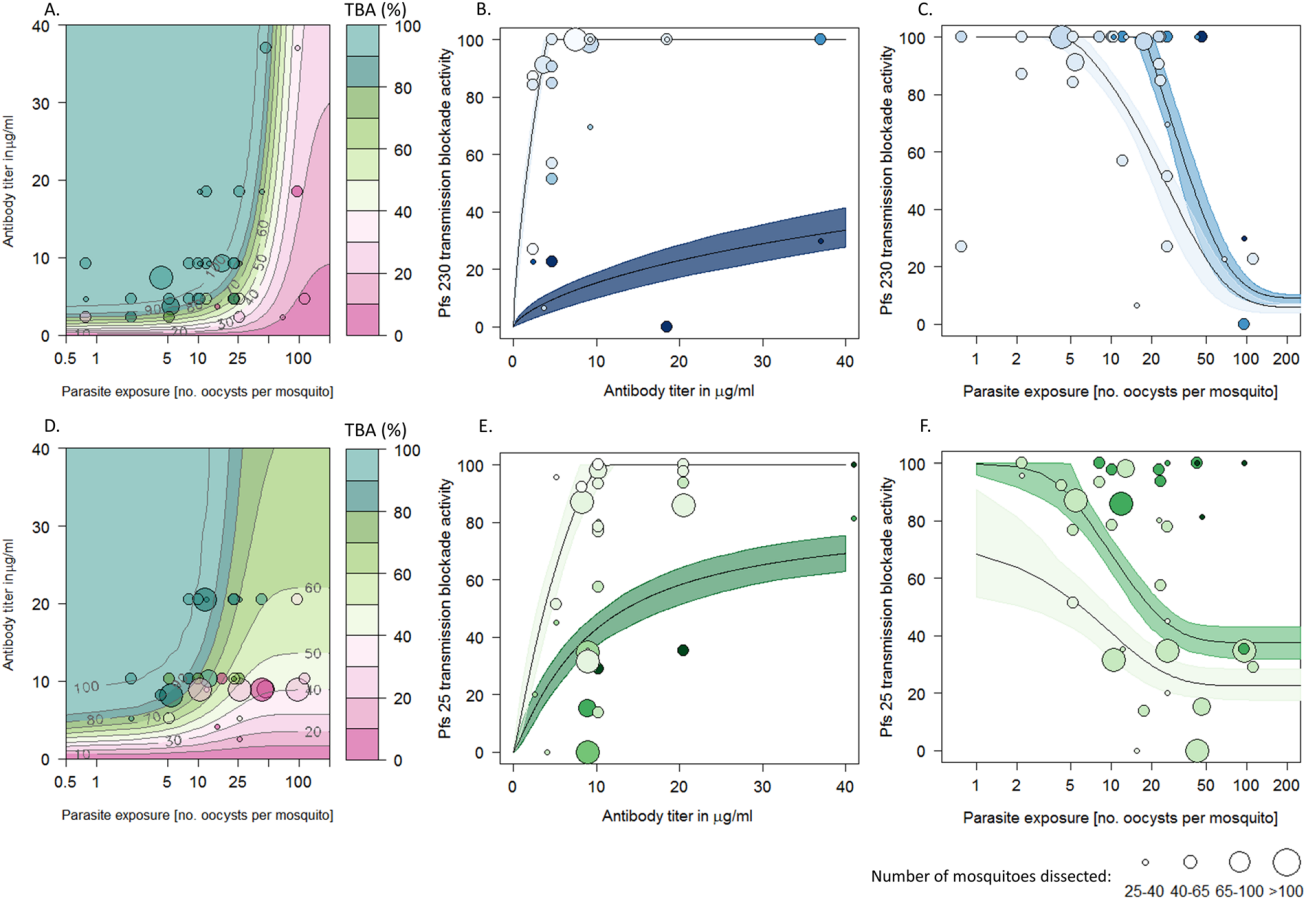
A comparison of transmission blocking activity (percentage reduction in the number of infected mosquitoes, TBA) for anti-Pfs230 (ABC) and anti-Pfs25 (DEF) antibodies. In all panels point size is proportional to the number of mosquitoes dissected. Panel (A) and (D): 3D model predictions for TBA depending on antibody specific titer and parasite exposure (as measured by the mean number of oocyst in the control group). Contour lines and colors show the best fit model for TBA which can be compared to the observed estimates (colors match contour plot). Panels (B) and (E) show the same model on a 2 dimensional plane illustrating how TBA varies with antibody titer. Solid lines indicating the best fit model whilst shaded regions show 95% confidence intervals around the best fit lines. The color of the shaded regions and points indicates the level of parasite exposure (darkest shades match highest exposures: for the two lines light shading = 5 oocysts/mosquito and dark shading = 100 oocysts/ mosquito). Panel (C) and (F): The same model showing how TBA changes with parasite exposure (best fit model with 95% confidence intervals) for two antibody titers. Point and area shading denote antibody titer (darkest shades match highest titers: light shade and dark shade respectively show model predictions for low (4 µg/ml) and high (40 µg/ml) titers).

The transmission blocking efficacy of anti-Pfs230-C and anti-Pfs25 antibodies varies with antibody titer and parasite exposure (Fig. 1). TBA increases with antibody specific titer though titer alone is a poor predictor of TBA. For example for anti-Pfs25 estimates of TBA from individual hosts for a titer of ~8 µg/ml varied from 38.2% (CI 32.6–43.6%) to 99.8% (CI 97.7–100%). Including information on parasite exposure significantly improved the predictive ability of the model (Supplementary information B compares the DIC values of all nested models tested). TBA was negatively correlated with parasite exposure so a higher antibody titer is required to block transmission in mosquitoes with a higher parasite exposure.

**Figure 2:**
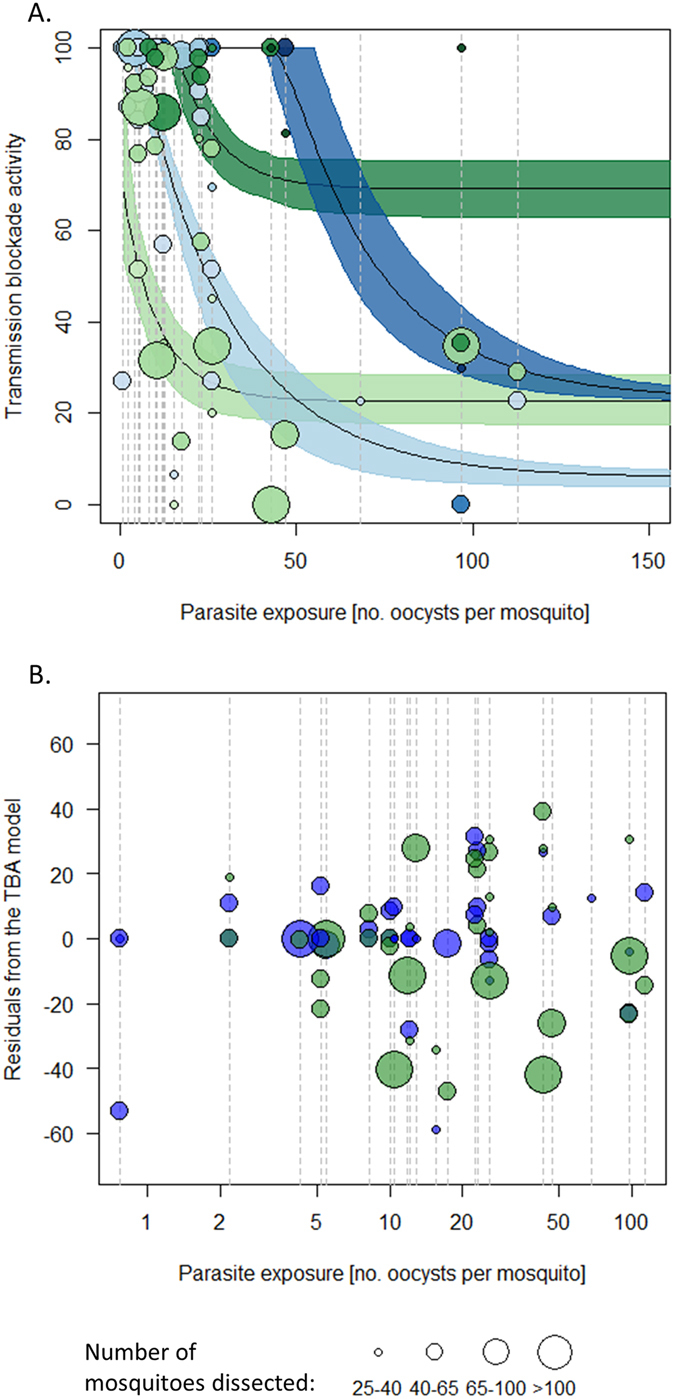
(**A**) An illustration of how transmission blocking activity (TBA) varies with parasite exposure for the two antibodies under investigation and (**B**). Residuals of the TBA model for experiments testing anti-Pfs230 (blue) and anti-Pfs25 (green) antibodies. In both panels vertical dashed line indicate assays run using the same parasite isolate (infected blood donated from an individual volunteer); green and blue colors indicate experiments testing TBA of anti-Pfs25 and anti-Pfs230 antibodies respectively. In (**A**), predictions for two titers are given, for 4 µg/ml of target-specific IgG (light colors) and 40 µg/ml of target-specific IgG (dark colors). Point shading indicates antibody titer. Care should be taken when interpreting the difference between the lines as both are fit using relatively few data points (especially at high levels of parasite exposure). In (**B**), each data point shows the distance from the observed data to the best fit model (taking into account differences in titer and exposure). A value of zero would indicate the model fitted the data perfectly. The symmetrically of the points either side of the line for each blood donor indicates that it is not always the parasite isolate having higher or lower TBA than predicted by the model.

The relationship between titer and TBA is highly non-linear with TBA increasing with titre before reaching a plateau. There is a significant difference in the 3D relationship between titer, parasite exposure and TBA depending on the target antigens, with anti-Pfs230-C TBA declines quicker with increasing parasite exposure (see Supplementary information B). The best fit model suggests that for antibodies targeting Pfs25 increasing titer can overcome extremely high parasite exposures (Fig. 2A). This pattern is not seen in anti-Pfs230, where increasing antibody titer above 11.1 µg/ml does not have a noticeable impact, though more experiments with high exposure will be required to confirm this result (Fig. 1B,E).

The model was then used to predict the minimum titer required to block transmission from hosts with different parasite exposures (Table 1). The range of parasite exposures seen in the field is unclear though most wild caught mosquitoes have relatively low numbers of oocysts (mean 5, range 1–105^16^). For all levels of parasite exposure tested (1–20 oocysts per mosquito), and for the polyclonal antibodies generated for these experiments, the anti-Pfs230-C antibodies can achieve a target efficacy of 50%, 80% or 99% efficacy with half the target-specific antibody concentration necessary for the anti-Pfs25 antibodies. This table is provided to show how the method can be used to directly compare different candidates which may have been fed on different blood-sources. As the avidity of human and mice antibodies may vary further experiments using the same analytical framework are required to select the best candidate for future clinical trials.

**Table 1:** Concentration of antigen-specific IgG necessary to achieve 99%, 80% and 50% target TBA with anti-Pfs230 and anti-Pfs25 antibodies, in hosts with different levels of parasite exposures: 1 oocysts/mosquitoes (low), 5 oocysts/mosquito (medium), 20 oocysts/mosquito (high).

			Target TBA		
			99% TBA	80% TBA	50% TBA
Parasite exposure	1	Anti-Pfs230	3.29 µg/ml	2.33 µg/ml	1.18 µg/ml
		Anti-Pfs25	7.50 µg/ml	4.89 µg/ml	2.81 µg/ml
	5	Anti-Pfs230	4.07 µg/ml	2.89 µg/ml	1.45 µg/ml
		Anti-Pfs25	10.37 µg/ml	6.89 µg/ml	3.75 µg/ml
	20	Anti-Pfs230	9.54 µg/ml	6.43 µg/ml	3.14 µg/ml
		Anti-Pfs25	/	23.19 µg/ml	8.18 µg/ml

This table is intended to show how future human antibodies can be directly compared as avidity between mouse and human antibodies may vary.

Even after taking into account antibody type, titer and parasite exposure, TBA is still highly variable (Fig. 1). Multiple feeds are done using the same parasite-source, i.e. blood from the same volunteer, to test different titers and different antibodies. This enables to assess whether parasite and/or host factors other than those captured in parasite exposure influence antibody efficacy (for example, is TBA higher when using one blood-source compared to another?). To statistically test this, the differences between the observed data and model predictions (the residuals) are calculated for both antibodies (Fig. 2B). The blood source represents only 17% of the residuals’ variance suggesting that variability around model predictions originates from noise in the experiment rather than infected blood from the same volunteer consistently over- or under-performing (paired rank sum test, p-value = 0.83). There was also no difference in the residual distribution between the tested antibodies (paired rank sum test, p-value = 0.84) indicating that the impact of the antibodies are consistent across parasite isolates. Figure 2B also shows that there is no evidence that the precision of TBA estimates changes with parasite exposure (*i.e*. the variance appears homoscedastic). This was confirmed with linear regression of residual-variance against parasite exposure or antibody titer (linear models, p-value = 0.63 and 0.75 respectively).

### Transmission-reducing efficacies of anti-Pfs230-C and anti-Pfs25

**Figure 3:**
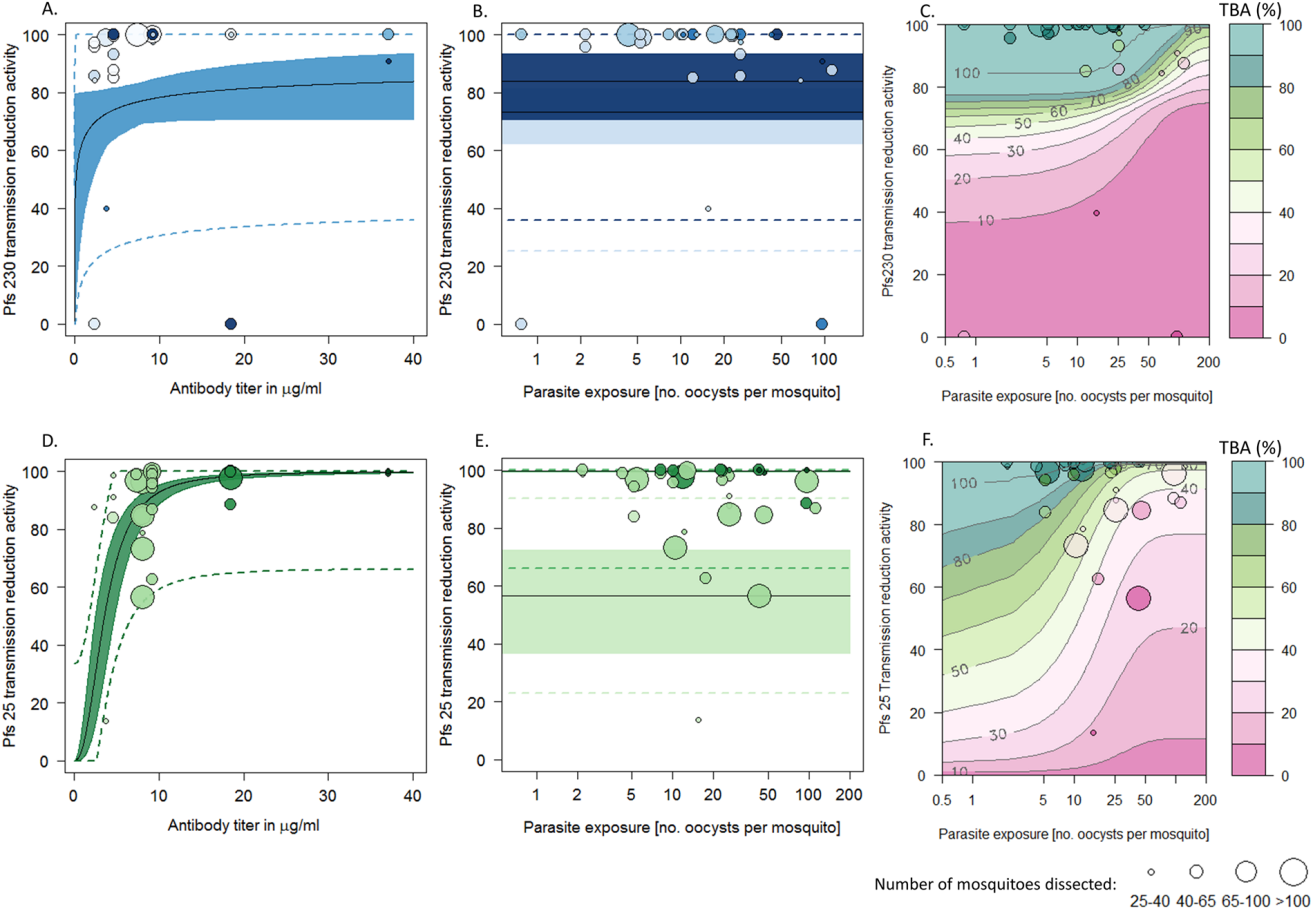
A comparison of transmission reduction activity (reduction in mean oocyst intensity, TRA) for anti-Pfs230 (panels A, B and C) and anti-Pfs25 (panels D, E and F) antibodies. In all panels point shading denotes exposure in oocysts/mosquito (darkest shades match highest exposures). Point size indicates the mean number of dissected mosquitoes dissected. (**A** and **D**) Predicted best fit relationship between TRA and antibody titer with 95% confidence intervals showing the uncertainty around the best fit line. The dashed line shows the 95% confidence intervals for single TRA estimates. B and E: Best fit model showing (with 95% confidence intervals) how TRA does not change with parasite exposure. Point and area shading feature antibody titer (dark colors match for 40 µg/ml IgG titers and light colors, for 4 µg/ml). (**C** and **F**) Model predictions of how transmission blocking efficacy (TBA) changes with TRA and parasite exposure. Contours show how the best fit models in Fig. 1 can be used to convert TRA into TBA according to the level of exposure. These model predictions are then compared to the true TBA results (data points).

TRA also increases with antibody titer for anti-Pfs230-C and anti-Pfs25 antibodies though, unlike TBA, there is no evidence to indicate that it varies with parasite exposure (Fig. 3, see Supplementary information B for DIC values). The shape of the relationship between titer and TRA is specific to each antibody, with anti-Pfs230-C reaching 83.5% (70.8–93.1) efficacy and anti-Pfs25 reaching 99.7% (99.3–99.8) efficacy. The accuracy of a single TRA estimate is relatively uncertain, as shown by the 95% confidence intervals for single TRA experiments (dashed lines in Fig. 3, panels A, B, D and E). The best fit model can be used to convert estimates of TRA into predictions of TBA according to the level of parasite exposure (2 C and F). This enables the efficacy of the intervention to be predicted for different settings using TRA data.

### Oocyst distribution in control and treatment mosquitoes

**Figure 4:**
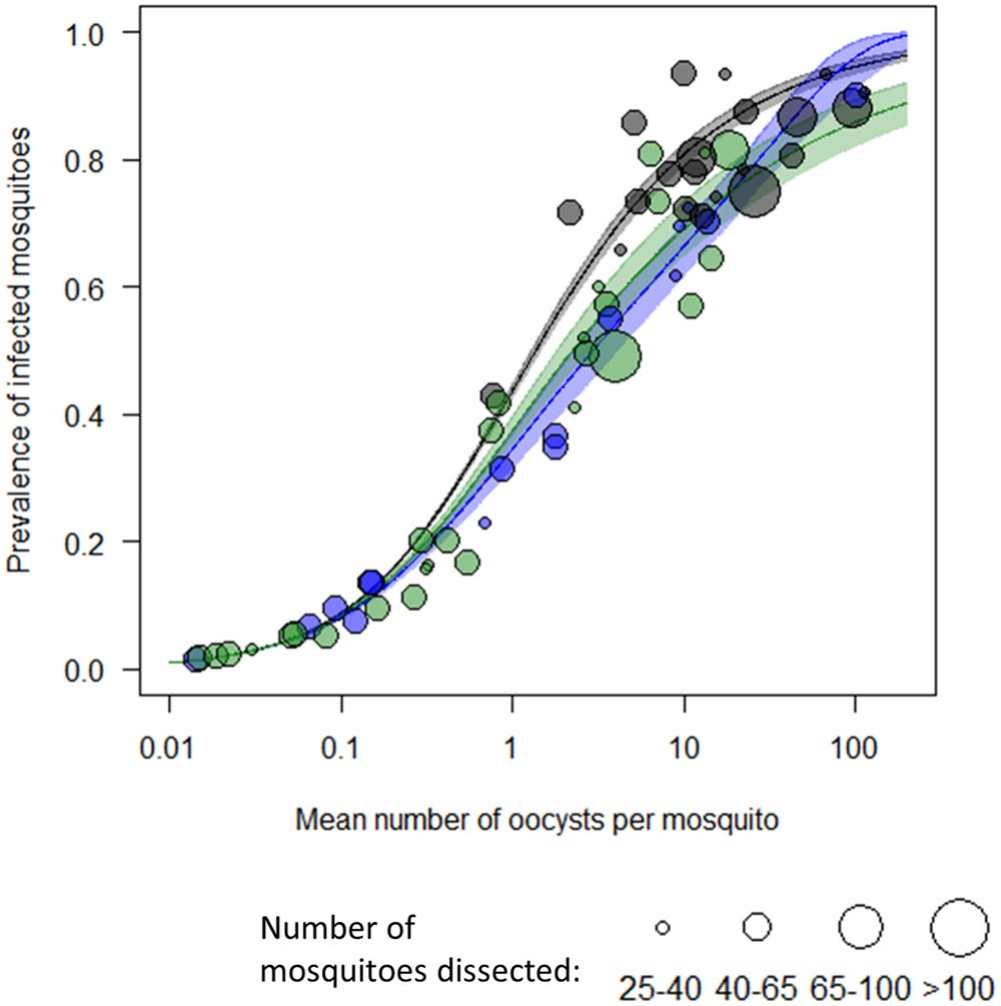
Prevalence of infected mosquitoes depending on the average number of oocysts per mosquitoes for the control group (black), for anti-Pfs230 (blue) and anti-Pfs25 (green). Solid line shows the best fit model for the relationship between prevalence and intensity of oocysts in the mosquito population as predicted by the model (shaded area shows 95% confidence intervals around the best fit line). Size of the data points are relative to the number of mosquitoes dissected.

The distribution of oocysts varies between the mosquitoes fed on infectious blood with control antibodies and mosquitoes fed on the same blood samples with SSM-VIMT antibodies and also between the groups of mosquitoes fed with different SSM-VIMT antibodies (Fig. 4, see Supplementary information B for DIC values). Even once the effect of oocysts intensity has been accounted for, overdispersion of oocysts is greater in the control group than in the treatments, and slightly greater in presence of anti-Pfs230-C than anti-Pfs25 antibodies. For example, the best fit curve indicates that at a mean of 10 oocysts per mosquito, 80% of oocysts in the control group are in 32.4% of fed mosquitoes whilst in those given anti-Pfs25, 80% of oocysts are in 23% of fed mosquitoes.

## Discussion

Pathogen dose is thought to be an important determinant of vaccine efficacy though empirical evidence of its importance is lacking^17^.The efficacy of two leading malaria SSM-VIMT candidates antibodies (Pfs25 and Pfs230) is dependent on parasite exposure with both antibodies being more effective the lower the mosquito forces of infection. This density dependency has previously been observed in the rodent model of malaria^18^ and using laboratory cultured parasites^5^ but this is the first time it has been demonstrated with naturally occurring parasites isolates in a local mosquito colony and in regards to antibody titer.

It is technically difficult to standardise parasite exposure so a direct comparison of TBA for two candidate SSM-VIMTs would require both antibodies to be tested in mosquitoes fed on the same parasite-source. The framework outlined here overcomes this requirement and will allow different SSM-VIMT candidates evaluated in different experiments (and potentially between laboratories) to be directly compared. This statistical method is also highly valuable in the context of DMFA experiments, as it allows to account for their inherent variability without overfitting the data. The analysis confirms that TRA is independent of parasite exposure from naturally occurring parasite isolates, making it the simplest metric for candidate screening and prioritization. As a general prediction, based on the results from anti-Pfs25 and anti-Pfs230, a SSM-VIMT which has an >80% TRA efficacy will on average have a >80% TBA efficacy assuming mosquito parasite exposure is <10 oocysts/midgut. The relationship between TRA and TBA appears to vary between antibodies which may be because the distribution of oocysts in groups of mosquitoes varies between the antibodies tested. This indicates that though TRA estimates may be sufficient for initial candidate screening^5^ they shouldn’t be used to predict the impact of expensive clinical trials where more tailored antibody-specific statistical models will be required.

It was previously demonstrated that the vast majority of low oocyst infections result in sporozoite positive mosquitoes and that oocyst density in the mosquito gut is positively associated with the number of sporozoites that reach the salivary gland^3^. It is currently assumed that all mosquitoes with salivary gland sporozoites are equally infectious irrespective of the number of parasites they harbour^19–21^. Recent work using rodent parasites indicates that mosquitoes with more salivary gland sporozoites are more likely to transmit the disease^22^. If this is the case for human malaria then the overall efficacy of a SSM-VIMT will depend on both TBA and TRA, as a vaccine which reduces the intensity of mosquito infection but fails to prevent salivary gland sporozoites may still reduce transmission. Nevertheless, until the impact of parasite intensity on mosquito-to-human transmission can be thoroughly quantified, TBA should remain the most epidemiologically important method of evaluating SSM-VIMT.

The high measurement error seen in TBA and TRA estimates means that individual MFA results should be treated with caution, and data from multiple assays and blood-sources should always be used. A framework such as the one outlined here can help to combine these assays. To provide less uncertainty around model predictions, each candidate should be evaluated at a broad range of titers and against a range of mosquito parasite exposures representative of the settings a SSM-VIMT would be deployed in. The experiment presented here use antibodies generated from immunized mice and direct extrapolation to the relationships between titer and efficacy for human antibodies will be hindered as the avidity of human and mice antibodies is likely to vary^23^. Similarly, other immunological factors such as complement and the interaction with Fc receptors may differ between humans and rodents. Studies of SSM-VIMT antibodies generated from human immunization are still limited in numbers and only target the antigen Pfs25, which so far has shown moderate TBA and lower than expected IgG concentrations^24–26^. In the future, the accumulation of TBA and TRA data generated from human SSM-VIMT antibodies will allow the 3D relationship between human antibody titre, parasite exposure and TBA to be fully characterised.

Understanding of how vaccine efficacy changes with parasite exposure will have important implications for how SSM-VIMT could be used to eliminate malaria. The average number of oocysts in wild caught mosquitoes typically lower than that seen in laboratories where SSM-VIMT candidates are initially evaluated^27^. This is encouraging as it indicates that the initial efficacy of a vaccine in the field might be higher than that observed in the laboratory. The distribution of oocysts in wild caught mosquitoes is also thought to be highly over-dispersed with most infected mosquitoes having only one or two oocysts whilst a few mosquitoes develop a high numbers^16, 28–30^. For example, a study carried out in the same village in Burkina Faso showed that mosquitoes which had detectable oocysts had a mean of 5.0 oocysts per mosquito with a range stretching from 1 to 105^16^. This indicates that at a population level, antibodies such as the ones targeting Pfs25 and Pfs230 might be highly effective, as they are likely to block transmission from the majority of recipients and only have partial efficacy on the rare occasion when a mosquito feeds on a highly infectious gametocyte carrier. It is likely that some of the oocysts in wild caught mosquitoes will have been acquired over multiple blood-meals^16^ so further work will be needed to estimate the parasite exposure per blood-meal and how this varies across the mosquito population. This will enable the framework developed here to predict the population efficacy of SSM-VIMT candidates in the field. It will also allow estimates of the minimum antibody titre required to eliminate transmission in a given location.

Population estimates of the efficacy of SSM-VIMT would allow to incorporate this strategy into transmission dynamics mathematical models and enable the mid and long-term public health impact of different SSM-VIMT candidates to be predicted. To date, it is unclear how parasite exposure changes as malaria is successfully controlled. If the exposure experienced by wild mosquitoes feeding on an infectious individual diminishes as malaria prevalence falls and antibody titer can be maintained, then SSM-VIMTs could become more efficacious over time. This would act in a positive feedback loop as the more the vaccine is effective at reducing transmission the more efficacious it becomes. This is supported by recent work which indicated that mosquitoes with fewer sporozoites are less infectious^22^. Nevertheless the processes governing human infectiousness are complex: a recent study Dielmo, Senegal, showed that there had not been an appreciable decline in the percentage of mosquitoes infected despite microscopically detected malaria prevalence in humans drastically fell^31^. Therefore, deciphering the relationship between malaria prevalence in human and parasite load in mosquitoes remains crucial for predicting the evolution of SSM-VIMT efficacy as malaria is successfully controlled. If SSM-VIMT efficacy does increase as malaria declines it would substantially enhance their utility in the push for malaria elimination.

## Materials and Methods

### Data

#### Membrane Feeding Assay

Direct membrane feeding assays (DMFAs) were carried out to using *Plasmodium falciparum* infected blood from a malaria endemic locality in Burkina Faso. Children 5–11 years old from the villages surrounding Bobo-Dioulasso were screened to check for the presence of *Plasmodium falciparum* gametocytes alone by microscopy after obtaining their parent/guardian’s informed consent. Venous blood samples drawn in heparinized tubes were centrifuged immediately after collection at 3,000 rpm for 3 minutes to remove the supernatant, which was replaced by non-immune serum from a European AB + donor. Total IgG (containing TBV induced antibody) purified from vaccinated mice were mixed with the donor blood samples. Two to three day old females from an outbred *Anopheles coluzzii* local colony were starved overnight and fed on the blood mixture through pre-warmed membrane feeders for 30 minutes. Fully fed females were sorted and maintained in cages at 28 °C ± 2, 80% ± 05 RH, with 10% glucose solution available. Mosquitoes were dissected day 7 post-feeding in a drop 0.5% mercurochrome and their midguts examined for oocysts by light microscopy. Individual efficacy estimates are generated by comparing the reduction in oocyst prevalence (TBA) and oocyst intensity (TRA) from the same parasite-sources with and without SSM-VIMT antigen. Altogether blood from 21 donors were used to test the efficacy of 6 different titers of each antibody with over 5000 mosquitoes being dissected in total. The titers, blood sources an number of dissected mosquitoes for all DMFA experiments that were carried out are detailed in Supplementary information C. All methods were carried out in accordance with relevant guidelines and regulations and the experimental protocol was approved by Burkina Faso Ethics Committee for Health Research (CERS).

### SSM-VIMT candidates

The lead vaccine candidate antigens (Pfs25 and Pfs230-C) and a control antigen, the green fluorescent protein (GFP), were expressed in viral vectors and their immunogenicity assessed in an 8 week prime-boost immunization of Balb/C mice^32^. Total IgG was purified from pooled serum obtained from all vaccinated mice with either viral-vectors expressing Pfs25 or Pfs230-C. Antibody titer was assessed by measuring total IgG. To allow a direct comparison between the two SSM-VIMT candidates IgG specific to Pfs25 and Pfs230-C was measured using the biacore method. This indicated that 8.2% of total IgG was specific to Pfs25 and 7.4% to Pfs230-C. These values were used to convert overall IgG to antibody specific titers that are used through the paper. All animal procedures to raise vaccine-specific responses were performed in accordance with Animal Scientific Procedures Act, United Kingdom, and approved by the University of Oxford Animal Care and Ethical Approval Committee.

### Statistical analysis

The overall impact of parasite exposure and antigen titer on the transmission reduction and transmission blocking efficacy of anti-Pfs25 and anti-Pfs230-C by fitting best fit curves to individual parasite count data using Markov Chains Monte-Carlo methods. Three distinct statistical models were fit: (1) a TBA model describing how the intervention reduces mosquito infection (oocyst prevalence) according to antibody titer and parasite exposure; (2) a TRA model describing how the intervention changes mean oocyst intensity dependent on antibody titer and parasite exposure; and (3) a prevalence-intensity model showing how the relationship between oocyst prevalence and intensity changes between control mosquitoes and the different antibodies. Oocyst prevalence is assumed to follow a binomial distribution whilst oocyst intensity is described by a negative binomial distribution. Detailed models can be found in Supplementary Information, Supplementary Information A. A variety of different functional forms were tested to determine the shape of the best fit relationship. Best fit functions were selected by comparing the Deviance Information Criterion, DIC, for a wide range of functions of varying complexity (from linear to second order polynomial, also including classical exponential, logarithmic and sigmoid functions). The DIC informs on the distance between model predictions and experimental data and penalize unnecessarily complex models; the smallest DIC number indicates the best fit function, with 2–3 DIC points’ difference being considered significant^33^. A detailed description of the best fit model and alternative good fit functions are available in Supplementary information B. Numerical simulation was done using a Gibbs Markov chain Monte Carlo sampling algorithm implemented in OpenBUGS^34^. Three Markov chains were initialized to assess convergence. All parameters were considered independents, and non-informative priors were employed. A total of 15,000 iterations were used (the first 10,000 being discarded) to derive the posterior distribution of parameters and generate 95% Bayesian credible interval estimates for best fit lines. Point estimates for TBA and TRA (and their associated uncertainty) for individual experiments (a single comparison of control and intervention mosquitoes infected using the same blood-source) were generated using generalised linear models^4^. Residuals from the TBA and TRA models for each experiment were studied using Wilcoxon rank sum test (single test for normality and paired test for antibody comparison) and linear mixed models with host as a random effect, to identify the impact of the host on the residual’s variance. Residuals where shown to be normally distributed justifying this structural assumption in the model. We also computed host performance as the sum of treatment residuals for each host on any given antibody, to detect if individual variability leads to systematically over- or under-performing when treated.

## Electronic supplementary material

Supplementary Information
