# Supplementary material for: Evaluation of two lead malaria transmission blocking vaccine candidate antibodies in natural parasite-vector combinations
Source: Sci Rep. 2017 Jul 28;7:6766. doi: 10.1038/s41598-017-06130-1 (PMC5533793; doi:10.1038/s41598-017-06130-1)
Supplement: Supplementary file 1 — Supplementary Information [file 41598_2017_6130_MOESM1_ESM.pdf]

Supplementary Information: Evaluation of two lead malaria transmission blocking vaccine candidate antibodies in natural parasite-vector combinations

Anais Bompard[1†*], Dari F. Da[2,3†], Serge Yerbanga[2], Sumi Biswas[4], Melissa Kapulu[4], Teun Bousema[5], Thierry Lefèvre[2,3,6], Anna Cohuet[3], Thomas S. Churcher[1]

[1] MRC Centre for Outbreak Analysis and Modelling, Infectious Disease Epidemiology, Imperial College London, Norfolk Place, London W2 1PG, UK.

[2] Institut de Recherche en Sciences de la Santé, Bobo-Dioulasso, Burkina Faso

[3] Unité MIVEGEC, IRD 224-CNRS 5290-Université Montpellier, Montpellier, France

[4] The Jenner Institute, University of Oxford

[5] Department of Medical Microbiology, Nijmegen, The Netherlands

[6] Laboratoire mixte international sur les vecteurs (LAMIVECT), Bobo Dioulasso, Burkina Faso.

[†] these authors contributed equally to the work

Supplementary Information A: Models

1. Prevalence-intensity model

The prevalence-intensity model links mean oocyst counts to the prevalence of oocysts in the mosquito population for each batch of mosquitoes. This provides a measure of the degree of parasite aggregation and explains the relationship between TBA and TRA [1,2].

Let i indicates the intervention group under investigation (be it 0=control mosquitoes, 1=anti-Pfs 25 or 2=anti-Pfs 230-C test antibodies) and j be the batch of mosquitoes fed on the same parasite-source and maintained together. The mean number of oocysts in a mosquito population (the intensity) in batch j given intervention i is denoted M_{ij} and is described by a negative binomial distribution with parameters $\propto_{ij}$ (constant success probability) and k_{ij} (a function describing the over-dispersion parameter,

$$M_{ij} = \frac{(1-\propto_{ij})k_{ij}}{\propto_{ij}}. \quad [1]$$

The number of blood-fed mosquitoes dissected is denoted N_{ij} and P_{ij} is the proportion of these with identifiable oocysts (the prevalence). The relationship between prevalence and intensity for data with a negative binomial distribution is given by the following equation,

$$P_{ij} = \left(1 - \frac{M_{ij}}{k_{ij}}\right)^{-k_{ij}}. \quad [2]$$

Allowing k_{ij} to vary (as a constant or dependent on mean parasite intensity) changes the shape of the relationship between oocyst prevalence and intensity. Here the full model uses a simple linear function,

$$k_{ij} = \alpha_i + \beta_i M_{ij} \ . \qquad [3]$$

It is assumed that the number of mosquitoes infected, denoted Y_{ij} is described by a binomial distribution then parameters α_i and β_i can be estimated for each treatment group using the following equation,

$$Y_{ij} \sim Binomial\ (P_{ij}, N_{ij}). \qquad [4]$$

Data from all intervention groups are fit at the same time allowing models with and without antibody specific α_i and β_i parameters to be directly compared. Models setting $\beta_i = 0$ were also run to determine whether the degree of overdispersion changed with parasite intensity.

2. Transmission-blocking activity model.

The transmission blocking activity of intervention i is defined by the percentage reduction in the prevalence of oocysts and is denoted E_i^P. For each treatment (i) and blood-source (j),

$$P_{ij} = P_{0j}(1 - E_{ij}^P). \qquad [5]$$

Transmission blockade is then decomposed into two functions capturing the impact of antibody titre (titre effect, T_i) and parasite exposure (exposure effect, C_j):

$$E_i^P = T_i \times C_j \qquad [6]$$

The relationship between titre and vaccine efficacy is typically described using the Hill equation,

$$T_i = \frac{(t_i/\mu_t)^{\gamma_1}}{(t_i/\mu_t)^{\gamma_1} + \gamma_2} \qquad [7]$$

where t_i is the antibody specific titre used of intervention i, μ_t is the mean titre on the experiment (a constant used to center the data and help the fitting process), and γ_{1-3} are parameters to be fitted. Equation [7] is compared to four simpler functions, a constant model where efficacy is independent of titre ($T_i = \gamma_1$) and a linear model ($T_i = \gamma_2 + \gamma_1 M_{ij}$), a simple exponential function ($T_i = 1 - \exp(-\gamma_1 M_{ij})$), and a sigmoid function ($T_i = 1/(1 + \exp(-\gamma_1 M_{ij} + \gamma_2)$). To determine whether functions varied between antibodies models using common or discrete parameter between antibodies were compared.

Following visual inspection of data TBA appears to decline at an approximate exponential rate with increasing parasite exposure (as defined as the mean oocyst intensity in the control group of mosquitoes from the same blood-source, M_{0j}). A variety of different functional forms were tested for the relationship and the full equation is given below,

$$C_j = \delta_1 + (1 - \delta_1)\exp\big(-(M_{0j} + \delta_2)\,\delta_3\big). \qquad [8]$$

Parameters δ_{1-3} are estimated from the fitting process. Setting these parameters to zero or one reduces Equation 8 to simpler (nested) functions which were fit and compared to ensure the most parsimonious model. The different titre effect and exposure effect models were compared against one another (a full list of the models tested is given in Supplementary information B). Equations [1] to [8] were fit to the full dataset simultaneously to enable the uncertainty in prevalence estimates (both control and intervention) and intensity estimates (in the control group only) to be accounted for in the best fit model and propagated in the uncertainty around the best fit line.

3. Transmission reduction activity model.

The transmission reducing efficacy (E_i^I) is defined as the ability to reduce the mean oocyst intensity in the mosquito population. It is estimated using the same set of mathematical functions used to estimate TBA (i.e. Equation [6]-[8], though substituting E_i^I for E_i^P in Equation [6]) though operates on the mean oocyst intensity,

$$M_{ij} = M_{0j}(1 - E_{ij}^I). \quad [9]$$

The model is fit to the individual oocyst data assuming a negative binomial distribution (using the same k_{ij} as described in Equation [3]). Models with separate intensity and titre effects for each antibody (with all different functional forms) are compared to those where a universal relationship is assumed. Fitting showed that adding an exposure effect did not improve the accuracy of the model. As for the transmission blockade model, Hill's function (eq. [7]) was best to describe the effect of titre on efficacy,

$$T_i = \frac{(t_i/\mu_t)^{\gamma'_1}}{(t_i/\mu_t)^{\gamma'_1} + \gamma'_2} \quad [10]$$

Full details of all models tested are shown in Supplementary information B.

4. Predicting TBA from TRA and parasite exposure

Transmission reduction efficacy can provide prediction of transmission blockade efficacy for each antibody according to the level of parasite exposure. Rearranging the best fit functions (see Supplementary information B for DIC) provides the following relationship,

$$TBA = \frac{\left(^{TRA\,\gamma'_2}/_{(1-TRA)}\right)^{\gamma_1/\gamma'_1}}{\left(^{TRA\,\gamma'_2}/_{(1-TRA)}\right)^{\gamma_1/\gamma'_1} + \gamma_2} \times C_j. \quad [11]$$

which generates TBA from TRA and intensity, where C_j is the exposure effect described in equation [8] and parameters γ'_1, γ'_2 and γ_1, γ_2 are obtained by fitting respectively TRA and TBA titre effect functions (see equation[7]). In the best fit models, these parameters are distinct for anti-Pfs 25 and anti-Pfs 230-C antibodies, which indicate that the shape of the relationship between TRA and TBA is specific to each antibody.

Supplementary information B. Model selection (DIC tables)

1. Transmission-blocking activity model

TBA Model		Titre effect				
		γ_1	$\gamma_2 + \gamma_1 M_{ij}$	$1 - \exp(-\gamma_1 M_{ij})$	$\frac{1}{1+\exp\begin{pmatrix}-\gamma_1 M_{ij} \\ +\gamma_2\end{pmatrix}}$	Hill's (equ. [7])
Exposure effect	δ_1	10420*	9576*	8485*	8429	8449*
	$\exp(-M_{0j}\,\delta_3)$	9215*	8159*	8272*	8144*	8112*
	$\exp(-(M_{0j}+\delta_2)\,\delta_3)$	8273*	NC	NC	8047	7905*
	$\delta_1 + (1-\delta_1)\exp(-(M_{0j}+\delta_2)\,\delta_3)$	8273*	NC	NC	NC	**7869**†

Supplementary table 1. Deviance Information Criteria (DIC) table for the transmission blocking activity model for the main functions tested for both the impact of parasite exposure (exposure effect) and titre concentration (titre effect). Superscripts indicate the best fit model for each functional relationship with the lowest DIC, be it one with separate titre and exposure effect functions for each antibody (*), separate titre and intensity functions apart from at least one parameter (δ_1 in best fit model) (†), constant titre and intensity functions when no sign are given. NC indicate that convergence could not be obtain with that combination of functions. Bold number represent the best fit functions for the model. For the best model the fit was better with separate exposure covariates (with common exposure covariate for anti-Pfs25 and anti-Pfs230-C DIC=8142 with distinct exposures covariates DIC=7869) and separates titre covariates apart for δ_1 parameter (common titer covariate for anti-Pfs25 and anti-Pfs230-C DIC=7900, with distinct titer covariates DIC=7869)

2. Transmission reduction activity model

TRA Model		Titre effect		
		γ'_1	$1-\exp(-\gamma'_1 M_{ij})$	Hill's (equ. [10])
Intensity effect	δ'_1	12670*	12590*	**12570***
	$\exp(-M_{0j}\,\delta'_3)$	12680†	12590†	12570†

Supplementary table 2. Deviance Information Criteria (DIC) table for the transmission blocking activity model for the main functions tested for both the impact of parasite exposure (exposure effect) and titre concentration (titre effect). Superscript indicates the model which gave the lowest DIC, be it one with separate titre and exposure effect functions for each antibody (*) or constant exposure effect but separate titre effects (†). Bold number represent the best fit functions for the model. The most parsimonious model had separate titre covariates (with common exposure covariate for anti-Pfs25 and anti-Pfs230-C DIC=12580 with distinct exposures covariates DIC= 12570).

3. Prevalence-intensity model

PI model		Group		
		All together	Treated *vs.* untreated	Antibodies separated
Overdispersion parameter	α_i	12470	12450	12430
	$\alpha_i+\beta_i M_{ij}$	12460	12450	12450
	Pfs 230-C: α_i<br>Pfs 25 : $\alpha_i+\beta_i M_{ij}$	/	12450	**12410**

Supplementary table 3. Deviance Information Criteria (DIC) table for the prevalence-intensity model for the main functions tested for both the impact of parasite exposure (exposure effect) and titre concentration (titre effect). Bold number represent the best fit functions for the model in this case a distinct, constant overdispersion parameter for control and anti-Pfs 25 and a linear overdispersion parameter for anti-Pfs 230-C.

Supplementary information C. Experiments tables

	Titers (µg/ml)	Nb. of blood sources	Nb. of mosquitoes dissected	Parasite prevalence in control groups	Parasite exposure
**Pfs 230-C experiments**	31.25	5	223	73.77±19.33	20.40±17.60
	62.50	11	512	77.73±13.65	18.15±9.25
	125	10	473	77.14±14.46	15.87±9.00
	250	5	219	79.14±5.98	35.54±41.57
	500	2	83	87.21±0.93	73.01±107.10
**Total Pfs 230-C**		20	1720	77.18±12.90	25.54±11.02
**Pfs 25 experiments**	31.25	1	35	75.11	25.91±50.36
	62.5	3	127	77.50±7.32	18.81±24.09
	109	6	659	80.37±6.14	37.10±36.08
	125	10	536	82.42±8.69	21.60±11.14
	250	9	475	82.02±6.08	31.77±27.80
	500	3	98	84.96±4.01	63.97±75.32
**Total Pfs 25**		19	2102	80.57±7.25	31.96±13.19
**Controls**		21	1604	78.89±10.77	28.65±7.44
**Total**		21	5426	78.89±10.77	28.65±7.44

Supplementary table 4. Table of DMFA experiments. Titers are in total IgG – the antibodies specific titer used in the paper represent 7.4% of total IgG for anti-Pfs230-C and 8.2% of total IgG for anti-Pfs-25. Parasite exposure is the average oocyst count in mosquitoes fed on the corresponding blood sources without treatment, as defined in the paper.

**Blood source Nb.**	Gametocytemia (gametocytes/µl of blood)	Parasite exposure (Nb. of oocysts/ mosquito)	**Blood source Nb.**	Gametocytemia (gametocytes/µl of blood)	Parasite exposure (Nb. of oocysts/ mosquito)
1	240	46.76±51.50	11	200	68.06±44.84
2	120	42.79±55.97	12	80	5.46±6.80
3	1752	96.68±203.06	13	192	12.84±21.46
4	80	25.91±50.36	14	80	4.29±5.25
5	144	12.16±12.05	15	168	23.21±19.34
6	32	0.78±1.12	16	48	2.18±2.74
7	120	15.45±15.48	17	48	5.21±5.58
8	136	11.81±14.34	18	120	22.46±21.66
9	168	17.40±12.52	19	136	8.20±9.16
10	224	112.90±74.05	20	112	10.07±7.79
			21	120	10.40±14.97

Supplementary table 5. Table of blood sources for DMFA experiments. Gametocytemia (in gametocytes per µl of blood) and parasite exposure (average oocyst count in mosquitoes fed on the blood source without treatment ± standard deviation) for each of the 21 blood sources used for the DMFA experiments.

Supplementary information D. Parameter table

(A) Transmission Blocking Activity Model			
	Description	Value	Code notation
μ_t	Average titre in realised experiments	154	
γ_1	Hills' coefficient in titre effect function (equ. [7])	Pfs 230: 0.71 (0.60-0.91)<br>Pfs 25: 1.17 (0.91-1.4)	k[2]<br>k[8]
γ_2	Apparent dissociation constant from titre effect Hill's function (equ. [7])	Pfs 230: 6.30 (5.79-7.5)	k[9]
δ_3	Exponential function parameter for exposure effect function (equ. [8])	Pfs 230: 0.04 (0.03-0.05)<br>Pfs 25: 0.10 (0.07-0.15)	k[1]<br>k[3]
δ_2	Exponential function parameter for intensity effect function (equ. [8])	Pfs 230: 4.52 (4.17-4.85)<br>Pfs 25: 2.33 (1.84-2.96)	k[11]<br>k[10]
δ_1	Distribution parameter for intensity effect function (equ. [8])	0.82 (0.77-0.86)	k[12]

(B) Transmission Reduction Activity Model			
	Description	Value	Code notation
γ'_1	Hills coefficient (equ. [10])	Pfs 230: 0.31 (0 - 0.75)<br>Pfs 25: 2.50 (2.08-2.97)	k[10]<br>k[6]
γ'_2	Apparent dissociation constant from Hill's function (equ. [10])	Pfs 230: 0.27 (0.18-0.39)<br>Pfs 25: 0.06 (0.04-0.09)	k[1]<br>k[9]

(C) Prevalence-Intensity Model			
	Description	Value	Code notation
α	Overdispersion parameter (equ. [3])	Control: 0.56 (0.57-0.52)<br>Pfs 25: 0.34 (0.30-0.35)<br>Pfs 230: 0.28 (0.22-0.29)	k[6]<br>k[8]<br>k[7]
β	Overdispersion parameter (equ. [3])	Pfs 230: 0.0036 (0.0016-0.0039)	k[1]

Supplementary table 6. Tables of parameters with brief description and best fit estimates for transmission blockade activity model (A) the transmission reduction activity model (B) and the prevalence-intensity model (C). The code notation indicates the different parameters in the OPENBUGS code used to fit the model (as presented in Supplementary information D).

Supplementary information E. Openbugs code

1. TBA model

```
model{
   for (j in 1:n_code) ##Number of hosts
          {
          no_pos_c[j]~dbin(my_pc[j],no_diss_c[j])
          my_pc[j]~dunif(0,1)
        for (i in code_offset[j]:code_offset[j+1]-1)
##Number of experiments (one titre and antibody per experiment)
          {
          no_pos_e[i]~dbin(my_pe[i],no_diss_e[i])
          my_pe[i]<-min(1,(my_pc[j])*(1-my_eff[i]))
## Prevalence after treatment fitted to prevalence before treatment and efficacy
          my_eff[i]<- is_treat[i]*min(1,my_eff_fun[i]*my_exp[i]) ## TBA
          my_eff_fun[i]<- (AB[i]-1)*(pow(my_titre[i]/mean(my_titre[]),k[8])
          /(pow(my_titre[i]/mean(my_titre[]), k[8])+k[7]))+(2-
          AB[i])*(pow(my_titre[i]/mean(my_titre[]) ,k[2])/(pow(my_titre[i]/mean(my_titre[]),
          k[2])+k[9])) ##Titre effect
          my_exp[i]<-(AB[i]-1)*(k[12]+(1-k[12])*exp(-mean_rand[j]*k[3]+k[10]))+(2-
          AB[i])*(k[12]+(1-k[12])*exp(-mean_rand[j]*k[1]+k[11])) ##Intensity effect
          }
          for (l in code_offset2[j]:code_offset2[j+1]-1)
          {
          my_rand[l]~dnegbin(a[j],b[j]) ###negative binomial distribution describes data
          }
          a[j]~dbeta(k[4],k[5])
          b[j]<- k[6] ###overdispersion parameter
          mean_rand[j]<-(1-a[j])*b[j]/a[j]
}
k[1]~dgamma(0.1,0.0001)
k[2]~dnorm(0.1,0.0001)
k[3]~dgamma(0.01,0.001)
k[4]~dgamma(0.1,0.001)
k[5]~dgamma(0.1,0.001)
k[6]~dgamma(0.1,0.001)I(0.00001,)
k[7]~dnorm(0.1,0.0001)
k[8]~dnorm(0.1,0.001)
k[9]~dnorm(0.1,0.001)
k[10]~dnorm(0.1,0.0001)
k[11]~dnorm(0.1,0.0001)
k[12]~dgamma(0.1,0.0001)I(,1)
}
```

2. TRA model

```
model{
for (j in 1:n_code)
	{
		for (l in code_offset2[j]:code_offset2[j+1]-1)
		{
		my_rand[l]~dnegbin(a[j],overdis[j])
		}
	a[j]~dbeta(k[4],k[5])
	mean_rand[j]<-(1-a[j])*overdis[j]/a[j]
	no_pos_c[j]~dbin(my_plc[j],no_diss_c[j])
	my_plc[j]<-min(1, max(0,1-pow((1+mean_rand[j]/overdis[j]),-overdis[j])))
				overdis[j]<-max(0.0001,k[2]+my_random[j])
	for (i in code_offset[j]:code_offset[j+1]-1)
		{
		overdisp_treat[i]<-max(0.0001,(AB[i]-1)*(k[8])+(2-
		AB[i])*(k[7]+k[3]*mean_rand_treated[i]))
		no_pos_e[i]~dbin(my_ple[i],no_diss_e[i])
		my_ple[i]<-min(1, max(0,1-pow((1+mean_treated[i]/overdisp_treat[i]),-
		overdisp_treat[i])))

			for (l in code_offset3[i]:code_offset3[i+1]-1)
			{
			my_rand_treated[l]~dnegbin(c[i],overdisp_treat[i])
			}
		mean_rand_treated[i]<-
mean(my_rand_treated[code_offset3[i]:(code_offset3[i+1]-1)])
		c[i]<-min(1,max(0.00001,1/(mean_treated[i]/overdisp_treat[i]+1)))
		mean_treated[i]<-(1-my_eff[i])*(mean_rand[j])
		my_eff[i]<- min(1, max(0, my_eff_fun[i]*my_exp[i]))
		my_eff_fun[i]<- (AB[i]-1)*(pow(my_titre[i]/mean(my_titre[])
		,k[6])/(pow(my_titre[i]/mean(my_titre[]), k[6])+k[9]))+(2-AB[i])*1/(1+exp(-
my_titre[i]/mean(my_titre[])*k[10]+k[1]))
		my_exp[i]<-1
		}
	}
k[1]~dnorm(0.1,0.001)
k[2]~dgamma(0.1,0.001)
k[3]~dnorm(0.1,0.001)
k[4]~dgamma(0.1,0.001)
k[5]~dgamma(0.1,0.001)
k[6]~dgamma(0,0.01)
k[7]~dgamma(0.1,0.001)I(0.0001,)
k[8]~dgamma(0.1,0.001)I(0.0001,)
k[9]~dgamma(0.1,0.1)
k[10]~dgamma(0.1,0.001)}
```

3. PI model

```
model{
   for (j in 1:n_code)
          {
          for (l in code_offset2[j]:code_offset2[j+1]-1)
                    {
                    my_rand[l]~dnegbin(a[j],overdis[j])
                    }
          mean_rand[j]<-mean(my_rand[code_offset2[j]:(code_offset2[j+1]-1)])
          a[j]~dbeta(k[4],k[5])I(0.0001,0.999999)
          intensity_control[j]<-(1-a[j])*overdis[j]/a[j]
          no_pos_c[j]~dbin(my_plc[j],no_diss_c[j])
          my_plc[j]<-min(1, max(0,1-pow((1+intensity_control[j]/overdis[j]),-overdis[j])))
          overdis[j]<-max(0.000001,k[6])

          for (i in code_offset[j]:code_offset[j+1]-1)
                    {
                    overdisp_treat[i]<-max(0.000001,(AB[i]-1)*(k[8])+(2-
          AB[i])*(k[7]+mean_rand_treated[i]*k[1]))
                    no_pos_e[i]~dbin(my_ple[i],no_diss_e[i])
                    my_ple[i]<-min(1, max(0,1-pow((1+intensity_treated[i]/overdisp_treat[i]),-
overdisp_treat[i])))
                    for (l in code_offset3[i]:code_offset3[i+1]-1)
                              {
                              my_rand_treated[l]~dnegbin(c[i],overdis[j])
                              }
                    mean_rand_treated[i]<-
                    mean(my_rand_treated[code_offset3[i]:(code_offset3[i+1]-1)])
                    c[i]~dbeta(k[2],k[3])I(0.0001,0.999999)
                    intensity_treated[i]<-(1-c[i])*overdisp_treat[i]/c[i]
                    }
          }
k[1]~dnorm(0.1,0.01)
k[2]~dgamma(0.1,0.01)
k[3]~dgamma(0.1,0.01)I(0.0001,)
k[4]~dgamma(0.1,0.01)I(0.0001,)
k[5]~dgamma(0.1,0.1)I(0.0001,)
k[6]~dnorm(0.1,0.01)I(0.0001,)
k[7]~dnorm(0.1,0.01)
}
```
